# GoPrime: Development of an In Silico Framework to Predict the Performance of Real-Time PCR Primers and Probes Using Foot-and-Mouth Disease Virus as a Model

**DOI:** 10.3390/pathogens9040303

**Published:** 2020-04-20

**Authors:** Emma L A Howson, Richard J Orton, Valerie Mioulet, Tiziana Lembo, Donald P King, Veronica L Fowler

**Affiliations:** 1 The Pirbright Institute, Ash Road, Pirbright, Surrey GU24 0NF, UK; emma.howson@pirbright.ac.uk (E.LAH.); valerie.mioulet@pirbright.ac.uk (V.M.); veronica.fowler@ecoanimalhealth.com (V.LF.); 2 Institute of Biodiversity, Animal Health and Comparative Medicine, College of Medical, Veterinary & Life Sciences, Graham Kerr Building, University of Glasgow, Glasgow G12 8QQ, UK; richard.orton@glasgow.ac.uk (R.JO.); tiziana.lembo@glasgow.ac.uk (T.L.); 3 MRC-University of Glasgow Centre for Virus Research, University of Glasgow, Glasgow G61 1QH, UK

**Keywords:** PCR, foot-and-mouth disease, diagnostics, in silico modeling framework

## Abstract

Real-time PCR (rPCR) is a widely accepted diagnostic tool for the detection and quantification of nucleic acid targets. In order for these assays to achieve high sensitivity and specificity, primer and probe-template complementarity is essential; however, mismatches are often unavoidable and can result in false-negative results and errors in quantifying target sequences. Primer and probe sequences therefore require continual evaluation to ensure they remain fit for purpose. This paper describes the development of a linear model and associated computational tool (GoPrime) designed to predict the performance of rPCR primers and probes across multiple sequence data. Empirical data were generated using DNA oligonucleotides (n = 90) that systematically introduced variation in the primer and probe target regions of a diagnostic assay routinely used to detect foot-and-mouth disease virus (FMDV); an animal virus that exhibits a high degree of sequence variability. These assays revealed consistent impacts of patterns of substitutions in primer and probe-sites on rPCR cycle threshold (C_T_) and limit of detection (LOD). These data were used to populate GoPrime, which was subsequently used to predict rPCR results for DNA templates (n = 7) representing the natural sequence variability within FMDV. GoPrime was also applicable to other areas of the FMDV genome, with predictions for the likely targets of a FMDV-typing assay consistent with published experimental data. Although further work is required to improve these tools, including assessing the impact of primer-template mismatches in the reverse transcription step and the broader impact of mismatches for other assays, these data support the use of mathematical models for rapidly predicting the performance of rPCR primers and probes in silico.

## 1. Introduction

Real-time PCR (rPCR) has become an essential tool in molecular biology and is routinely used for detection, quantification, and differentiation of nucleic acids in both research and diagnostic settings [1,2,3]. Central to the specificity and sensitivity of rPCR assays are the primers and probes, with amplification affected by factors such as primer and probe-template complementarity and the presence of secondary structures (e.g., primer dimers) [4]. However, designing primers and probes with full sequence complementarity to all the required targets can be problematic. For instance, when considering RNA viruses such as foot-and-mouth disease virus (FMDV), the high mutation rate (in the range of 10^−3^ to 10^−5^ per nucleotide site, per genome replication [5,6]) can result in fully conserved regions being too short to accommodate primer and probe sets. This is especially true when designing assays to target the more varied genomic regions for serotype/strain differentiation [7,8,9,10]. Consequently, primer and probe-template mismatches are often unavoidable and a compromise approach that accommodates sequence mismatches is often adopted to design diagnostic tests. 

The effects of mismatches on PCR amplification have been well studied and quantified for both primers [11,12,13,14,15,16,17,18,19,20,21,22] and probes [20,21]. For instance, primer-template mismatches in the 3′-end region of the primer have been shown to have a larger effect on PCR amplification than those located towards the 5′-end, due to disruption of the DNA polymerase active site [11,16,18,20,22]. Furthermore, for rPCR probes, the position of mismatches in the oligonucleotide have been shown to differentially destabilize probe annealing [20].

Primer and probe-template mismatches can be especially problematic when considering the use of rPCR for diagnostic purposes. By impacting rPCR amplification, mismatches can alter the cycle threshold (C_T_) at which targets are detected, leading to errors in nucleic acid quantification. For instance, a single internally located mismatch can result in up to a 1000-fold underestimation of initial copy number [18]. Notably, mismatches at the 3′-end of primers have been shown to produce effects ranging from a two-fold underestimation of initial copy number to complete prevention of amplification, thus leading to false-negative results [22]. As such, false-negative rPCR results may be common, especially in the instance of assays detecting lower viral loads (for FMDV, this is common in oesophageal–pharyngeal fluid and environmental samples).

These effects of mismatches result in the requirement for continual evaluation of primer and probe sequences. In addition to laborious manual laboratory-based screening, primer and probe validation traditionally occurs though Basic Local Alignment Search Tool (BLAST) searches against publicly available sequences [23], with tools now developed to automate the process [24,25,26]. However, despite numerous studies into the effects of mismatches, no primer evaluation programs to date have been developed using experimental data, with target sequences only reported as putative hits or misses. With rPCR assays requiring different performance criteria depending upon their use, the provision of binary predictions is limited. For example, high specificity is paramount for assays used to differentiate between diseases, high sensitivity is required for assays used to confirm negative results and an awareness of cross-reactivity is important for assays that distinguish between closely related sequences, such as FMDV serotypes and viral lineages [9]. As such, the availability of a quantitative primer/probe validation program could support rPCR evaluation by giving researchers and diagnosticians the ability to rapidly predict whether assays are fit for purpose.

This paper describes the impacts of different primer and probe-template mismatches on C_T_ and limit of detection (LOD) on rPCR, and the presentation of a primer and probe evaluation framework (GoPrime), in order to ascertain to what extent the effect of mismatches on template cDNA can be predicted. Further analysis is required to assess the effects of primer-template mismatches during the reverse transcription step.

## 2. Materials and Methods

### 2.1. The Effects of Primer and Probe-Template Mismatches

For all experimental analyses, primer sequences were kept the same and the template sequences were varied. The primer and probe sequences, published by Callahan et al. (2002), were as follows: forward primer: 5′-ACT GGG TTT TAC AAA CCT GTG A-3′ (Tm: 56.5 °C); reverse primer, 5′-GCG AGT CCT GCC ACG GA-3′ (Tm: 60 °C); and probe 5′-(6FAM)TCC TTT GCA CGC CGT GGG AC(TAMRA)-3′ [27] (Tm calculated at www.eurofinsgenomics.eu/en/ecom/tools/oligo-analysis/). Linear DNA oligonucleotide templates of 109 bp (Sigma-Aldrich, Munich, Germany) were designed around the cDNA target region for a published assay [27]: a conserved region of the FMDV genome (3D_pol_-coding region). Ninety templates were ordered, each designed to evaluate the consequences of different variations in the primer or probe binding regions (Table 1). For example, variations across the length of the primer and probe target regions were designed to investigate the effect of position, with different bases substituted to study the effects of mismatch type and mismatch quantity. Sequences were based on FMDV O/UKG/35/2001 (accession number KR265074, nucleotides 7862-7970). In addition, a template with full primer/probe-template complementarity was ordered and used as the reference template (R) for all rPCR runs. 

### 2.2. Real-Time PCR

rPCR reactions were performed using two Taq-based rPCR kits.:

(1) Excite^TM^ UF 2x Master Mix (Excite^TM^ UF) (Quantig Ltd., Camberley, UK), a Taq-based rPCR kit, was selected as it required minimal reaction set-up, increasing the likelihood of assay variation being attributed to target sequence differences rather than human variability. Reactions were performed in a total of 20 µL, containing: 5 µL template, 10 µL 2x master mix, 50 nM ROX reference dye, 1.6 µL of each primer (16 pmol), 1.2 µL of probe (6 pmol) (primers and probes final concentrations as previously described [28]) and made up to volume with nuclease-free water (NFW). Thermal cycling conditions were 95 °C for 3 min, followed by 50 cycles of 95 °C for 5 s and 60 °C for 20 s.
(2) SuperScript™ III Platinum™ One-Step qRT-PCR Kit (SSIII^TM^) (Thermo Fisher Scientific, Waltham, MA, USA) was chosen as it is a commonly used Taq-based kit. Reagents, parameters, primer/probe final concentrations and thermal cycling conditions were as previously reported [28]. Reactions were performed in a total of 25 µL, containing: 5 µL template, 12.5 µL 2x buffer, 0.5 µL of Superscript III enzyme mix (both supplied with the kit), 50 nM ROX reference dye, 2 µL of each primer (20 pmol), 1.5 µL of probe (7.5 pmol) [27] and made up to volume with NFW. Thermal cycling conditions were 95 °C for 10 min, followed by 50 cycles of 95 °C for 15 s and 60 °C for 1 min. The reverse transcription (RT) step was omitted from the published protocol [28].

All rPCR reactions were performed on an ABI ViiA^TM^ 7 real-time PCR system thermocycler (Thermo Fisher Scientific). Positive reactions were defined as those that gave a detectable C_T_ (no C_T_ cut-off set). Initial rPCR reactions were performed in duplicate using 10^6^ copies of template. Where C_T_ values were detected, further rPCR reactions were performed in duplicate across a log_10_ dilution series of template (10^6^–10^0^ copies/reaction) in 0.1 µg/µL carrier RNA (Ambion®, Austin, TX, USA, Thermo Fisher Scientific, Waltham, MA, USA). The effect of mismatches on rPCR were determined by calculating the change in C_T_ (ΔC_T_) and change in LOD (ΔLOD) between the reference and varying oligonucleotide DNA templates.

### 2.3. Development of GoPrime

In order to ascertain the effect of each primer and probe-template mismatch type on ΔC_T_ and ΔLOD, linear model analysis was performed. Linear model variables (Table 2) were selected based on statistical analysis (to ascertain which primer and probe-template mismatch locations were statistically different from one another) and published data (Supplementary Data Table S1).

Linear model analysis [29] was performed in R [30], using the variables stated in Table 2, and all quantitative data collected (90 templates, all template dilutions, using both *Taq*-based kits, to analyze the average effects of mismatches [one linear model looked primer-template mismatches; a second linear model was used to look at probe-template mismatches]). The results of the linear models were used to parameterize GoPrime: a computational tool for predicting the effects of mismatches on rPCR. GoPrime was built by implementing the primer and probe mismatch rules and C_T_ penalties in a computer program written in the Java programming language. GoPrime takes as input fasta sequence files of the primers/probe and target sequences. It calculates an expected change in C_T_ for each target sequence set based on the number and type of mismatches between the primer/probe and template present. The GoPrime program is freely available from: https://github.com/rjorton/GoPrime.

### 2.4. Evaluating GoPrime as a Predictor of rPCR Performance

In order to evaluate GoPrime on naturally occurring sequence variations, a search was performed using the National Centre for Biotechnology Information (NCBI) nucleotide database (NCBI, 2017). Seven FMDV sequences, which contained naturally occurring variations in the Callahan et al. (2002) primer and probe target regions [27], were selected and ordered as linear DNA oligonucleotides of 109 bp (Table 3). These were used as template in rPCR, using both the Excite^TM^ UF and SSIII^TM^ protocols, with ΔC_T_ and ΔLOD results compared with predictions from GoPrime.

In order to ascertain how transferable GoPrime was to other areas of the FMDV genome, GoPrime was used to analyze four FMDV-typing assays designed to target the less conserved VP1/2A-coding regions [9]. The four sets of primers and probes (specific for either serotype A, O, Southern African Territories [SAT] 1 or SAT 2 field viruses circulating in East Africa) were evaluated against the 66 VP1/2A-coding sequences used in the initial laboratory-based evaluation (A = 15; O = 20; SAT 1 = 19; SAT 2 = 12). The published experimental results [9] and GoPrime predictions for the likely targets of each assay were then compared and displayed using GoPrimeTree. GoPrimeTree is a simple Java program that takes an existing nexus tree of the target sequences, and adds FigTree [31] readable coloring to the tree tips based on the GoPrime predicted ΔC_T_ values; different shades of the colors green, orange, and red were used to represent target sequences with ΔC_T_’s between 0–10, 10–20, and 20–30, respectively, whilst black was used for sequences that failed to amplify.

### 2.5. Statistical and Phylogenetic Analysis

Statistical analyses were performed using R [30]. Phylogenetic trees for the visualization of ΔC_T_ GoPrime results across FMDV serotypes were produced from sequence alignments in Mega (version 7.0.21) [32] using the neighbor-joining method [33] and viewed in FigTree (version 1.4.3) [31].

## 3. Results

### 3.1. The Effects of Primer/Probe-Template Mismatches on rPCR

**Figure 1:**
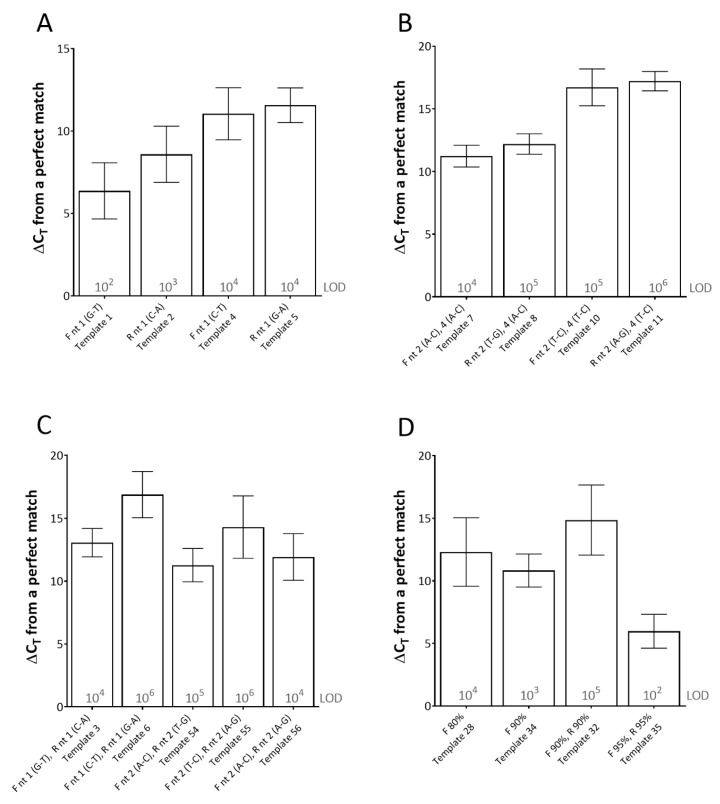
The effects of primer-template mismatches on real-time PCR (rPCR) cycle threshold (C_T_). (**A**) single mismatches at the 3′-end; (**B**) and (**C**) multiple mismatches at the 3′-end(s); (**D**) effect of primer-template percentage complementary. Results represent the average increase in cycle threshold (ΔC_T_) from the reference template across two rPCR kits (Excite^TM^ UF 2x Master and SuperScript™ III Platinum™ One-Step qRT-PCR Kit) and a dilution series of template (10^6^–10^0^ copies/reaction). The limit of detection (LOD) for each template is defined as the lowest dilution where all replicates were positive (displayed in grey text). Error bars represent the standard deviation. (F) forward primer; (R) reverse primer; (nt) nucleotide. Template number refers to Table 1.

For this assay, single mismatches between the template and the primer, at the 3′-end of the primer (using both rPCR kits) had the most detrimental effect on C_T_ (average ΔC_T_ of between 6.38 and 11.57 across the dilution series) (Figure 1). Furthermore, the type of mismatch was shown to be important. For instance, at the 3′-end of the reverse primer, a C-A mismatch (type 1 mismatch: purine-pyrimidine) resulted in an average effect of ΔC_T_ of 8.59 across the dilution series, whereas a G-A mismatch (type 2 mismatch: purine-purine or pyrimidine-pyrimidine) in the same location resulted in an average effect of ΔC_T_ of 11.57 across the dilution series (Figure 1A). Multiple mismatches in the 3′-end of the primers (either (i) both in one primer or (ii) one the forward and one in the reverse primer) either prevented amplification from occurring or required high template copy number (10^4^–10^6^ copies per reaction) for any amplification to occur, depending on the mismatches (Figure 1B,C).

**Figure 2:**
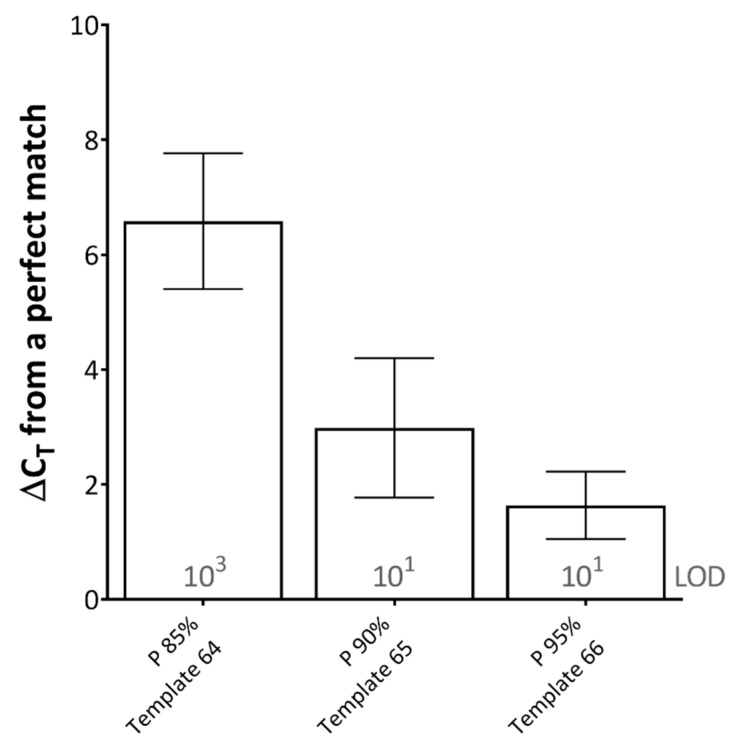
The effects of probe-template mismatches on real-time PCR (rPCR) cycle threshold. Results represent the average increase in cycle threshold (C_T_) from a perfectly matched template, across two rPCR kits (Excite^TM^ UF 2x Master and SuperScript™ III Platinum™ One-Step qRT-PCR Kit) and a dilution series of template (10^6^–10^0^ copies/reaction). The limit of detection (LOD) for each template is defined as the lowest dilution where all replicates were positive (displayed in grey text). Error bars represent the standard deviation. (P) probe; percentages represent probe-template complementarity. Template number refers to Table 1.

When studying the effect of primer-template percentage complementarity across the total length of the primers, a minimum of 82.05% primer-template match between the forward and reverse primers (combined) was required for amplification to occur (Figure 1D). When studying the effect of the mismatches across the length of the probe, a minimum of 85% probe-template complementarity was required in order for effective detection to occur, impacting upon rPCR ΔC_T_ by 6.58 on average across the dilution series (Figure 2). 

### 3.2. Development of GoPrime

Using linear model analysis, the average effect of each primer and probe-template mismatch type was determined, accounting for both single and multiple mismatches in the primer and probe binding regions, by implementing additive and dampening effects where necessary (Table 4). These results from the linear model, the minimum match percentages (82.05% primers combined, 85% probe), combined maximum of 2 mutations at the 3′ end of primers, and ΔC_T_ penalties for mutation types/combinations were used to parameterize the GoPrime program. 

**Figure 3:**
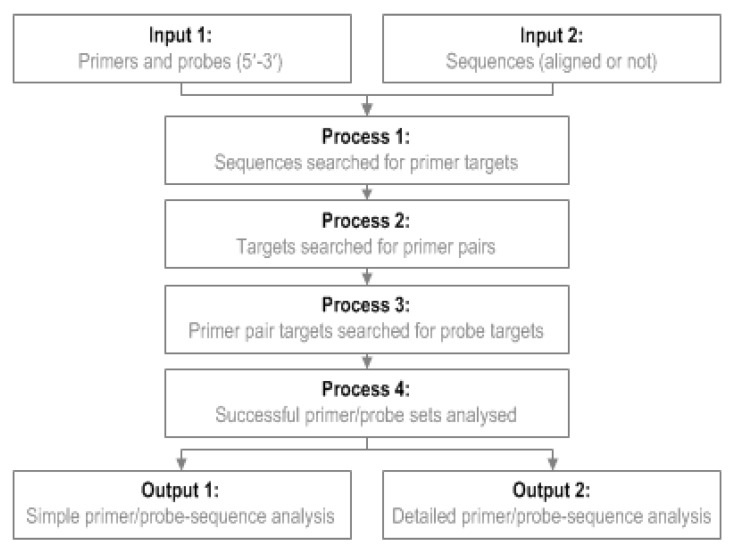
GoPrime flow diagram. GoPrime examines sets of primer sequences (optionally including a probe sequence), searches the target genome sequences for potential matches, then predicts the effect of any primers/probe-template mismatches present on real-time PCR cycle threshold and limit of detection.

To use GoPrime, users provide a file of their primer/probe sequences (5′-3′ fasta format) and a file of template sequences to be analyzed (5′-3′ fasta format). GoPrime first analyzes the template, in both orientations, for possible forward and reverse primer targets (Figure 3); at this stage, forward and reverse primers are analyzed individually to generate potential targets to investigate further as possible pairs. This is done based on the minimum requirements for primer-template complementarity, which were defined during data analysis (Table 4). Once possible forward and reverse primer targets are identified, they are evaluated as possible primer pairs (Figure 3). Potential probe targets (optional) are then identified between the primer pairs, searching again in both orientations based on the probe-template mismatch limits determined during data analysis (Table 4). 

Once a potentially suitable primer and probe set has been identified, GoPrime uses the parameters determined in the linear model to predict whether amplification is likely to occur and the effect of any mismatches present on ΔC_T_ and ΔLOD, implementing additive/dampening effects of multiple mismatches if applicable. GoPrime provides the outputs as two separate text files. Firstly, a simple analysis, which provides each sequence name against the predicted ΔC_T_ and ΔLOD, number of mismatches present and the likely amplicon length. The second analysis provides more detail, including the position and orientation of each likely primer/probe target and number and the type of any mismatches present (Figure 3, Supplementary Data Figure S2). 

If users have a corresponding phylogenetic tree of their target sequences, they can visualize the results by using the GoPrimeTree program which will color the tip labels of the tree based on the GoPrime ΔC_T_ results. In order to use this, users provide the text file output of GoPrime in addition to a phylogenetic tree (nexus format). GoPrimeTree color codes the sequences according to the predicted ΔC_T_, via annotation of the nexus tree file, so that the predicted targets and effect of the primer/probe-template mismatches can be easily visualized across multiple target sequences in FigTree [31].

### 3.3. Evaluating GoPrime as a Predictor of rPCR Performance

**Figure 4:**
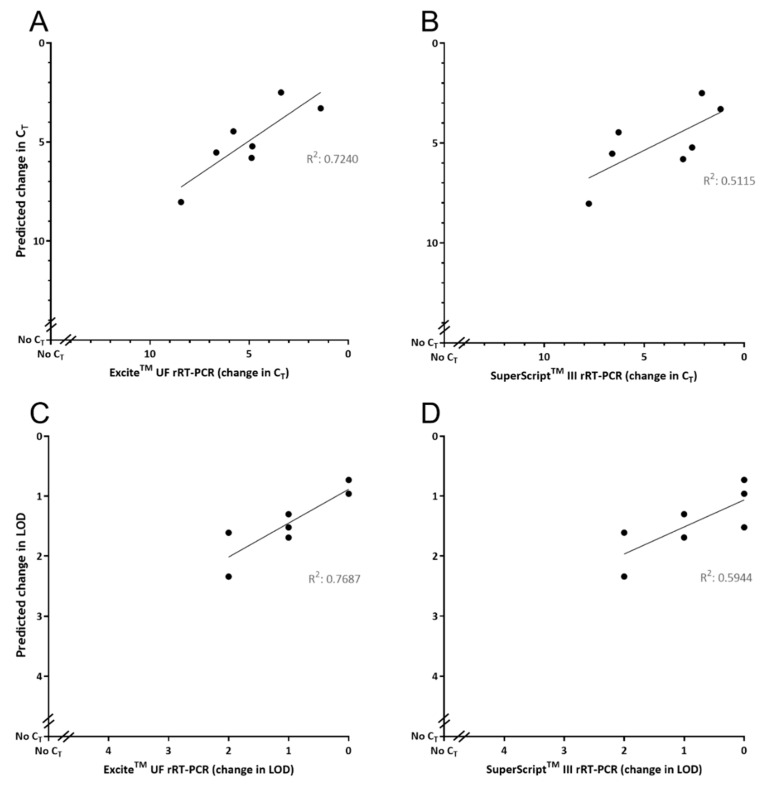
Evaluating GoPrime as a predictor of real-time PCR (rPCR) performance using naturally occurring sequence variations. GoPrime predictions are plotted against the (**A**) observed change in cycle threshold for Excite^TM^ UF 2x Master Mix; (**B**) observed change in cycle threshold for SuperScript™ III Platinum™ One-Step qRT-PCR Kit; (**C**) observed change in limit of detection for Excite^TM^ UF 2x Master Mix; (**D**) observed change in limit of detection for SuperScript™ III Platinum™ One-Step qRT-PCR Kit. For the observed results, points represent the average change in cycle threshold or limit of detection across all dilutions (10^6^–10^0^) of the starting template.

Evaluation of GoPrime using the seven DNA oligonucleotide templates containing sequence variations, observed in naturally occurring FMDV isolates in the Callahan et al. (2002) target region [27] (Table 3), showed that GoPrime on average predicted the ΔC_T_ of reactions 1.49 (SD 1.20; range 0.01–6.37) away from the observed result, when looking at all data points across the dilution series (Figure 4). In addition, GoPrime on average predicted the ΔLOD of reactions 0.63 (range 0.30–1.52) away from the observed result (Figure 4, Supplementary Data Table S3). 

**Figure 5:**
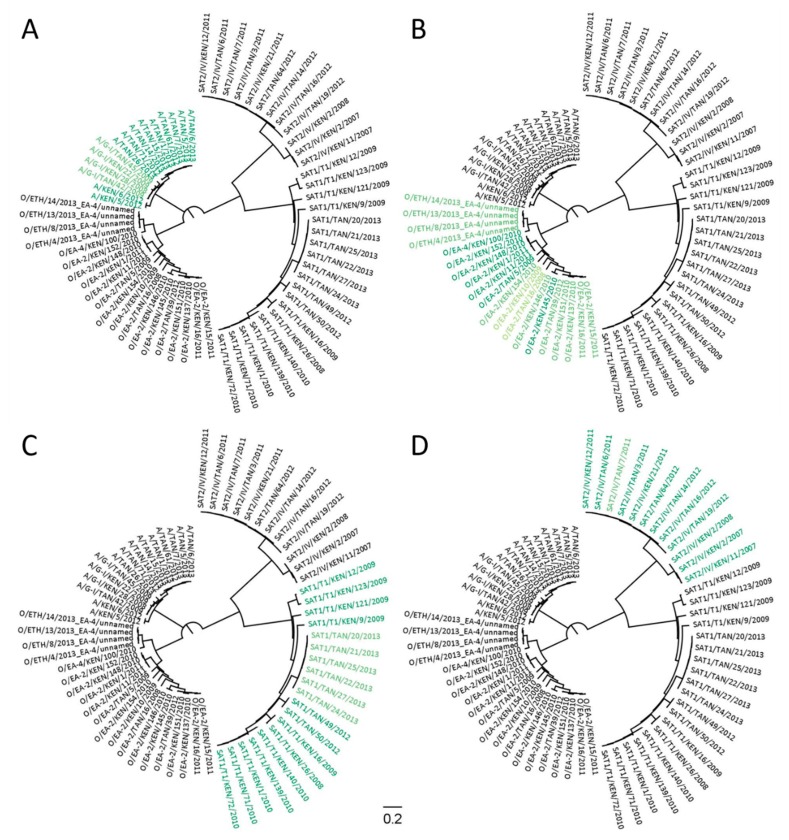
Using GoPrime and GoPrimeTree to predict the likely targets of foot-and-mouth disease virus (FMDV)-typing PCR assays (Bachanek-Bankowska et al., 2016) (n = 66). Four primer/probe sets were tested: (**A**) serotype A; (**B**) serotype O; (**C**) serotype Southern African Territories (SAT) 1; (**D**) serotype SAT 2. For the color scheme: (dark green) perfect primer/probe-template match; (mid-green) cycle threshold affected by up to a CT of 5; (light green) cycle threshold affected by up to a C_T_ of 10; (black) sequence predicted not to amplify.

Although GoPrime can only be currently used to evaluate rPCR and two-step rRT-PCR assays (in which RT and PCR stages are separate and mismatches persist through to cDNA), GoPrime was able to identify the likely targets for four FMDV-typing assays (East Africa specific for serotypes A, O, SAT 1, and SAT 2). The targets identified were consistent with previously published results, with each set of serotype-specific primers identifying the template sequences within their target serotype. However, cross-reactivity between serotypes was not predicted by GoPrime, which was evident in the published results [9] (Figure 5, Supplementary Data Figure S4).

## 4. Discussion

Primer and/or probe-template mismatches are often unavoidable in rPCR, leading to the requirement for continual monitoring of oligonucleotides used in assays against available sequence data, to ensure that assays remain fit for purpose. Consequently, the ability to quantitatively evaluate the performance of rPCR primers and probes in silico could aid researchers and diagnosticians by rapidly predicting the effects of mismatches present on rPCR amplification, which is not possible using current methods. 

The GoPrime framework is currently limited to rPCR (DNA template). For GoPrime to predict the effect of mismatches on one-step rRT-PCR, where gene-specific primers are used in both the RT and rPCR stages, further work is required to analyze the effect of mismatches between RNA templates and primers during the reverse transcription step. Although GoPrime should be applicable to two-step rRT-PCR, where the use of Oligo(dT) or random hexamers for the RT stage results in primer and probe-template mismatches persisting from RNA though to cDNA, further analysis is required to confirm this. Furthermore, our experimental design is currently limited to a single real-time PCR machine and two PCR kits. With inherent differences between PCR machines and due to different rRT-PCR kits having previously been shown to differ in their tolerance to mismatches [22], it would be beneficial to investigate how the general mismatch rules reported are affected by factors such as equipment performance and assay format.

Empirical data generated in this study were consistent with previous publications: mismatches in the 3′-end of primers had a more detrimental effect on rPCR amplification than those located towards the 5′-end, due to disruption of the DNA polymerase active site [11,16,18,20,22]. The effect of single mismatches within the 3′-end region displayed a consistent pattern, related to both nucleotide position and mismatch type. The effect of probe-template mismatches also displayed a consistent pattern, however, further testing and analysis on the positional effects of probe-template mismatches is required in order for these positional effects to be accurately included within GoPrime, in addition to probe-template percentage complementarity. For example, mismatches in the center of the probe have been previously reported to destabilize probe-template annealing [20]. At present, GoPrime splits mismatches into two types: (type 1) purine-pyrimidine mismatch (G-T or C-A nucleotide base pairing, leading to a minor conformational change in the primer/probe-template duplex) and (type 2) purine-purine or pyrimidine-pyrimidine mismatch (G-A, A-A, G-G, C-T, T-T or C-C nucleotide base pairing, leading to a major conformational change in the primer/probe-template duplex). Furthermore, the current model of GoPrime looks at the average effect of primer/probe-template mismatches across all template copy numbers. Future frameworks, to improve accuracy, could consider categorizing mismatches further and could include the effect of template copy number and PCR efficiency.

With rPCR assays requiring different performance criteria depending upon their use, the ability of GoPrime to quantitatively predict the effect of primer/probe-template mismatches on both C_T_ and LOD could help diagnosticians accurately assess whether rPCR assays are fit for their intended use. For instance, predicting whether assays that aim to differentiate between serotypes are specific to that serotype. Furthermore, some rPCR assays might give positive results in spite of mismatches when high viral loads are present (such as in acute stages of disease), but generate a false-negative in the presence of lower viral loads (such as in oesophageal–pharyngeal fluid or environmental samples for FMDV). Although the experimental data gained in this study was specific to the rPCR conditions and primer/probe sequences evaluated, GoPrime predicted the likely positive targets of four FMDV-typing assays, which target alternative regions of the FMDV genome. However, as cross-reactivity between serotypes was not predicted, the next stage for analysis would be to include ΔC_T_ data from other assays and to test the program across other organisms, to analyze GoPrime’s versatility and broaden the genomic context of the analysis. Furthermore, the analysis for GoPrime is currently based on data from synthesized DNA oligonucleotides diluted in carrier RNA. Further work is required to ensure that the general mismatch rules are consistent across clinical samples, which have more complex matrices including background nucleic acid and mixed DNA populations.

In conclusion, this paper describes the development of GoPrime: a freely available primer evaluation program which predicts the likely performance of primer/probe sets across multiple sequence data. Experimental data suggested that mismatch impacts follow a consistent pattern, enabling GoPrime to be parametrized from experimental observations. Within this study, GoPrime was only validated against primers and probes targeting FMDV, and a further research avenue would be to challenge GoPrime with alternative targets, to ascertain the broader prediction accuracy of this tool with additional assay targets. By providing a novel quantitative approach to primer/probe evaluation, GoPrime offers increased flexibility to the user by not only predicting the likely targets of primer/probe sets, but also estimating the effects of any mismatches present on C_T_ and LOD in silico, thereby enabling selection of the most appropriate primer/probe combination given the research question and diagnostic sample. 

## Figures and Tables

**Table 1:** Linear DNA oligonucleotide templates for real-time PCR targets (5′-3′).

	Forward Primer Target	Probe Target	Reverse Primer Target
R	ACTGGGTTTTACAAACCTGTGA	TCCTTTGCACGCCGTGGGAC	TCCGTGGCAGGACTCGC
1	ACTGGGTTTTACAAACCTGTG G	TCCTTTGCACGCCGTGGGAC	TCCGTGGCAGGACTCGC
2	ACTGGGTTTTACAAACCTGTGA	TCCTTTGCACGCCGTGGGAC	C CCGTGGCAGGACTCGC
3	ACTGGGTTTTACAAACCTGTG G	TCCTTTGCACGCCGTGGGAC	C CCGTGGCAGGACTCGC
4	ACTGGGTTTTACAAACCTGTG C	TCCTTTGCACGCCGTGGGAC	TCCGTGGCAGGACTCGC
5	ACTGGGTTTTACAAACCTGTGA	TCCTTTGCACGCCGTGGGAC	G CCGTGGCAGGACTCGC
6	ACTGGGTTTTACAAACCTGTG C	TCCTTTGCACGCCGTGGGAC	G CCGTGGCAGGACTCGC
7	ACTGGGTTTTACAAACCT A T A A	TCCTTTGCACGCCGTGGGAC	TCCGTGGCAGGACTCGC
8	ACTGGGTTTTACAAACCTGTGA	TCCTTTGCACGCCGTGGGAC	T T C A TGGCAGGACTCGC
9	ACTGGGTTTTACAAACCT A T A A	TCCTTTGCACGCCGTGGGAC	T T C A TGGCAGGACTCGC
10	ACTGGGTTTTACAAACCT T T T A	TCCTTTGCACGCCGTGGGAC	TCCGTGGCAGGACTCGC
11	ACTGGGTTTTACAAACCTGTGA	TCCTTTGCACGCCGTGGGAC	T A C T TGGCAGGACTCGC
12	ACTGGGTTTTACAAACCT T T T A	TCCTTTGCACGCCGTGGGAC	T A C T TGGCAGGACTCGC
13	ACTGG A TT C TAC G AAC T TGTGA	TCCTTTGCACGCCGTGGGAC	TCCGTGGCAGGACTCGC
14	ACTGGGTTTTACAAACCTGTGA	TCCTTTGCACGCCGTGGGAC	TCCGT A GC G GGACT T GC
15	ACTGG A TT C TAC G AAC T TGTGA	TCCTTTGCACGCCGTGGGAC	TCCGT A GC G GGACT T GC
16	ACTGG T TT G TAC C AAC A TGTGA	TCCTTTGCACGCCGTGGGAC	TCCGTGGCAGGACTCGC
17	ACTGGGTTTTACAAACCTGTGA	TCCTTTGCACGCCGTGGGAC	TCCGT T GC C GGACT A GC
18	ACTGG T TT G TAC C AAC A TGTGA	TCCTTTGCACGCCGTGGGAC	TCCGT T GC C GGACT A GC
19	A T T A G A TT C T G C G AAC T TGTGA	TCCTTTGCACGCCGTGGGAC	TCCGTGGCAGGACTCGC
20	ACTGGGTTTTACAAACCTGTGA	TCCTTTGCACGCCGTGGGAC	TCCGT A GC G GG G CT T G T
21	A T T A G A TT C T G C G AAC T TGTGA	TCCTTTGCACGCCGTGGGAC	TCCGT A GC G GG G CT T G T
22	A A T T G T TT G T C C C AAC A TGTGA	TCCTTTGCACGCCGTGGGAC	TCCGTGGCAGGACTCGC
23	ACTGGGTTTTACAAACCTGTGA	TCCTTTGCACGCCGTGGGAC	TCCGT T GC C GG C CT A G A
24	A A T T G T TT G T C C C AAC A TGTGA	TCCTTTGCACGCCGTGGGAC	TCCGT T GC C GG C CT A G A
25	A A T A G T TT C T C C G AAC A TGTGA	TCCTTTGCACGCCGTGGGAC	TCCGTGGCAGGACTCGC
26	ACTGGGTTTTACAAACCTGTGA	TCCTTTGCACGCCGTGGGAC	TCCGT T GC G GG C CT T G A
27	A A T A G T TT C T C C G AAC A TGTGA	TCCTTTGCACGCCGTGGGAC	TCCGT T GC G GG C CT T G A
28	ACTGG T TT C TAC G AAC A TGTGA	TCCTTTGCACGCCGTGGGAC	TCCGTGGCAGGACTCGC
29	ACTGGGTTTTACAAACCTGTGA	TCCTTTGCACGCCGTGGGAC	TCCGT T GC G GGACT T GC
30	ACTGG T TT C TAC G AAC A TGTGA	TCCTTTGCACGCCGTGGGAC	TCCGT T GC G GGACT T GC
31	ACTGG T TTTTAC G AAC A TGTGA	TCCTTTGCACGCCGTGGGAC	TCCGT T GC G GGACT T GC
32	ACTGG T TTTTACAAAC A TGTGA	TCCTTTGCACGCCGTGGGAC	TCCGT T GCAGGACT T GC
33	ACTGG T TTTTAC G AAC A TGTGA	TCCTTTGCACGCCGTGGGAC	TCCGT T GCAGGACTCGC
34	ACTGG T TTTTACAAAC A TGTGA	TCCTTTGCACGCCGTGGGAC	TCCGTGGCAGGACTCGC
35	ACTGGGTTTTACAAAC A TGTGA	TCCTTTGCACGCCGTGGGAC	TCCGT T GCAGGACTCGC
36	ACTGG T TT C TAC G AAC A TGTGA	TCCTTTGCACGCCGTGGGAC	C CCGTGGCAGGACTCGC
37	ACTGG T TT C TAC G AAC A TGTGA	TCCTTTGCACGCCGTGGGAC	G CCGTGGCAGGACTCGC
38	ACTGG T TT C TAC G AAC A TGTGA	TCCTTTGCACGCCGTGGGAC	T T CGTGGCAGGACTCGC
39	ACTGG T TT C TAC G AAC A TGTGA	TCCTTTGCACGCCGTGGGAC	T A CGTGGCAGGACTCGC
40	ACTGG T TTTTACAAAC A TGTGA	TCCTTTGCACGCCGTGGGAC	C CCGTGGCAGGACTCGC
28	ACTGG T TT C TAC G AAC A TGTGA	TCCTTTGCACGCCGTGGGAC	TCCGTGGCAGGACTCGC
29	ACTGGGTTTTACAAACCTGTGA	TCCTTTGCACGCCGTGGGAC	TCCGT T GC G GGACT T GC
30	ACTGG T TT C TAC G AAC A TGTGA	TCCTTTGCACGCCGTGGGAC	TCCGT T GC G GGACT T GC
31	ACTGG T TTTTAC G AAC A TGTGA	TCCTTTGCACGCCGTGGGAC	TCCGT T GC G GGACT T GC
32	ACTGG T TTTTACAAAC A TGTGA	TCCTTTGCACGCCGTGGGAC	TCCGT T GCAGGACT T GC
33	ACTGG T TTTTAC G AAC A TGTGA	TCCTTTGCACGCCGTGGGAC	TCCGT T GCAGGACTCGC
34	ACTGG T TTTTACAAAC A TGTGA	TCCTTTGCACGCCGTGGGAC	TCCGTGGCAGGACTCGC
35	ACTGGGTTTTACAAAC A TGTGA	TCCTTTGCACGCCGTGGGAC	TCCGT T GCAGGACTCGC
36	ACTGG T TT C TAC G AAC A TGTGA	TCCTTTGCACGCCGTGGGAC	C CCGTGGCAGGACTCGC
37	ACTGG T TT C TAC G AAC A TGTGA	TCCTTTGCACGCCGTGGGAC	G CCGTGGCAGGACTCGC
38	ACTGG T TT C TAC G AAC A TGTGA	TCCTTTGCACGCCGTGGGAC	T T CGTGGCAGGACTCGC
39	ACTGG T TT C TAC G AAC A TGTGA	TCCTTTGCACGCCGTGGGAC	T A CGTGGCAGGACTCGC
40	ACTGG T TTTTACAAAC A TGTGA	TCCTTTGCACGCCGTGGGAC	C CCGTGGCAGGACTCGC
41	ACTGG T TTTTACAAAC A TGTGA	TCCTTTGCACGCCGTGGGAC	G CCGTGGCAGGACTCGC
42	ACTGGGTTTTACAAACCTG A G G	TCCTTTGCACGCCGTGGGAC	TCCGTGGCAGGACTCGC
43	ACTGGGTTTTACAAACCTG C G G	TCCTTTGCACGCCGTGGGAC	TCCGTGGCAGGACTCGC
44	ACTGGGTTTTACAAACCTG A G C	TCCTTTGCACGCCGTGGGAC	TCCGTGGCAGGACTCGC
45	ACTGGGTTTTACAAACCTGT TG	TCCTTTGCACGCCGTGGGAC	TCCGTGGCAGGACTCGC
46	ACTGGGTTTTACAAACCTGT AG	TCCTTTGCACGCCGTGGGAC	TCCGTGGCAGGACTCGC
47	ACTGGGTTTTACAAACCTGT TC	TCCTTTGCACGCCGTGGGAC	TCCGTGGCAGGACTCGC
48	ACTGGGTTTTACAAACCTG GA A	TCCTTTGCACGCCGTGGGAC	TCCGTGGCAGGACTCGC
49	ACTGGGTTTTACAAACCTG CA A	TCCTTTGCACGCCGTGGGAC	TCCGTGGCAGGACTCGC
50	ACTGGGTTTTACAAACCTG GT A	TCCTTTGCACGCCGTGGGAC	TCCGTGGCAGGACTCGC
51	ACTGGGTTTTACAAACCT T TG G	TCCTTTGCACGCCGTGGGAC	TCCGTGGCAGGACTCGC
52	ACTGGGTTTTACAAACCT A TG G	TCCTTTGCACGCCGTGGGAC	TCCGTGGCAGGACTCGC
53	ACTGGGTTTTACAAACCT T TG C	TCCTTTGCACGCCGTGGGAC	TCCGTGGCAGGACTCGC
54	ACTGGGTTTTACAAACCTGT A A	TCCTTTGCACGCCGTGGGAC	T T CGTGGCAGGACTCGC
55	ACTGGGTTTTACAAACCTGT T A	TCCTTTGCACGCCGTGGGAC	T A CGTGGCAGGACTCGC
56	ACTGGGTTTTACAAACCTGT A A	TCCTTTGCACGCCGTGGGAC	T A CGTGGCAGGACTCGC
57	ACTGGGTTTTACAAACCT A T T A	TCCTTTGCACGCCGTGGGAC	TC T GTGGCAGGACTCGC
58	ACTGGGTTTTACAAACCT A T T A	TCCTTTGCACGCCGTGGGAC	TC A GTGGCAGGACTCGC
59	ACTGGGTTTTACAAACCT A T T A	TCCTTTGCACGCCGTGGGAC	C CCGTGGCAGGACTCGC
60	ACTGGGTTTTACAAACCT A T T A	TCCTTTGCACGCCGTGGGAC	G CCGTGGCAGGACTCGC
61	ACTGGGTTTTACAAACCTGTGA	TCCTT G GC G C A C A G C GG T AC	TCCGTGGCAGGACTCGC
62	ACTGGGTTTTACAAACCTGTGA	TCCTT G GC G C A CCG C GG T AC	TCCGTGGCAGGACTCGC
63	ACTGGGTTTTACAAACCTGTGA	TCCTT G GCAC A CCG C GG T AC	TCCGTGGCAGGACTCGC
64	ACTGGGTTTTACAAACCTGTGA	TCCTT G GCAC A CCG C GGGAC	TCCGTGGCAGGACTCGC
65	ACTGGGTTTTACAAACCTGTGA	TCCTTTGCAC A CCG C GGGAC	TCCGTGGCAGGACTCGC
66	ACTGGGTTTTACAAACCTGTGA	TCCTTTGCAC A CCGTGGGAC	TCCGTGGCAGGACTCGC
67	ACTGGGTTTTACAAACCTGTGA	C CCTTTGCACGCCGTGGGAC	TCCGTGGCAGGACTCGC
68	ACTGGGTTTTACAAACCTGTGA	G CCTTTGCACGCCGTGGGAC	TCCGTGGCAGGACTCGC
69	ACTGGGTTTTACAAACCTGTGA	TCCTTTGCACGCCGTGGGA T	TCCGTGGCAGGACTCGC
70	ACTGGGTTTTACAAACCTGTGA	TCCTTTGCACGCCGTGGGA A	TCCGTGGCAGGACTCGC
71	ACTGGGTTTTACAAACCTGTGA	GG CTTTGCACGCCGTGGGAC	TCCGTGGCAGGACTCGC
72	ACTGGGTTTTACAAACCTGTGA	TCCTTTGCACGCCGTGGG CT	TCCGTGGCAGGACTCGC
73	ACTGGGTTTTACAAACCTGTGA	C C A TTTGCACGCCGTGGGAC	TCCGTGGCAGGACTCGC
74	ACTGGGTTTTACAAACCTGTGA	TCCTTTGCACGCCGTGG T A T	TCCGTGGCAGGACTCGC
75	ACTGGGTTTTACAAACCTGTGA	T T C G TTGCACGCCGTGGGAC	TCCGTGGCAGGACTCGC
76	ACTGGGTTTTACAAACCTGTGA	TCCTTTGCACGCCGTG T G G C	TCCGTGGCAGGACTCGC
77	ACTGGGTTTTACAAACCTGTGA	C CCTTTGCAC A CCG C GGGAC	TCCGTGGCAGGACTCGC
78	ACTGGGTTTTACAAACCTGTGA	TCCTTTGCAC A CCG C GGGA T	TCCGTGGCAGGACTCGC
79	ACTGGGTTTTACAAACCTGTGA	CA CTTTGCACGCCGTGGGAC	TCCGTGGCAGGACTCGC
80	ACTGGGTTTTACAAACCTGTG G	TCCTT G GCAC A CCG C GGGAC	TCCGTGGCAGGACTCGC
81	ACTGGGTTTTACAAACCTGTG G	TCCTTTGCAC A CCG C GGGAC	TCCGTGGCAGGACTCGC
82	ACTGGGTTTTACAAACCTGTG C	TCCTTTGCAC A CCG C GGGAC	TCCGTGGCAGGACTCGC
83	ACTGGGTTTTACAAACCT T TG G	TCCTTTGCAC A CCG C GGGAC	TCCGTGGCAGGACTCGC
84	ACTGGGTTTTACAAACCTGTG G	C CCTTTGCACGCCGTGGGAC	TCCGTGGCAGGACTCGC
85	ACTGGGTTTTACAAACCTGTG G	TCCTTTGCACGCCGTGGGA T	TCCGTGGCAGGACTCGC
86	ACTGG T TTTTACAAAC A TGTGA	TCCTTTGCAC A CCG C GGGAC	TCCGT T GCAGGACT T GC
87	ACTGG T TTTTACAAAC A TGTGA	TCCTTTGCAC A CCG C GGGAC	TCCGTGGCAGGACTCGC
88	ACTGGGTTTTACAAAC A TGTGA	TCCTTTGCAC A CCGTGGGAC	TCCGT T GCAGGACTCGC
89	ACTGGGTTTTACAAAC A TGTGA	C CCTTTGCACGCCGTGGGAC	TCCGTGGCAGGACTCGC
90	ACTGGGTTTTACAAAC A TGTGA	TCCTTTGCACGCCGTGGGA T	TCCGTGGCAGGACTCGC
The primer/probe target sequences of the 90 DNA templates (109 base pairs in length) in 5′-3′ orientation. Non-target regions between the primer/probe targets were identical to O/UKG/35/2001 (accession number KR265074: nucleotides 7862-7970). The black sequence (top row) represents the reference template (R); grey sequences represent the varying DNA templates, black highlighted bases depict primer/probe-template mismatch sites.			

**Table 2:** Variables included in the linear model analysis.

Mismatch Type	Variable
Primers (forward or reverse)	Percentage mismatch (forward and reverse combined)
	Type 1 mismatch at the 3′-end (nucleotide 1)
	Type 2 mismatch at the 3′-end (nucleotide 1)
	Type 1 mismatch at the 3′-end (nucleotide 2)
	Type 2 mismatch at the 3′-end (nucleotide 2)
	Type 1 mismatch at the 3′-end (nucleotides 3-4)
	Type 2 mismatch at the 3′-end (nucleotides 3-4)
Probe	Percentage mismatch
Mismatches were grouped as one of two types: (type 1) purine-pyrimidine mismatch (G-T or C-A nucleotide base pairing, leading to a minor conformational change in the primer/probe-template duplex); (type 2) purine-purine or pyrimidine-pyrimidine mismatch (G-A, A-A, G-G, C-T, T-T or C-C nucleotide base pairing, leading to a major conformational change in the primer/probe-template duplex).	

**Table 3:** Linear DNA templates representing naturally occurring foot-and-mouth disease field isolates for testing GoPrime predictions (5′-3′).

	Forward Primer Target	Probe Target	Reverse Primer Target
R	ACTGGGTTTTACAAACCTGTGA	TCCTTTGCACGCCGTGGGAC	TCCGTGGCAGGACTCGC
JX040500	ACTGGGTTTTACAAACCT A TGA	TCCTTTGCACGCCGTGGGAC	TCCGTGGCAGGACTCGC
KC440884	ACTGG A TTTTA T AAACCTGTGA	TCCTTTGCACGCCGTGGGAC	TCCGTGGCAGGACTCGC
AY593802	ACTGGGTTTTACAAACCTGTGA	TCCTT C GCACGCCGTGGGAC	TC T GTGGCAGG G CTCGC
KC440883	ACTGGGTTTTACAAACCTGTGA	TCCTTTGCACGCCGTGGGAC	TC T GTGGC G GGACTCGC
AY593812	ACTGGGTTTTACAAACCTGTGA	TCCTTTGCACGCCGTGGGAC	TC A GTGGCAGGACTCGC
KF112882	ACTGGGTTTTACAAACCTGTGA	C CCTTTGCACGCCGTGGGAC	TCCGTGGCAGGACTCGC
HM191257	ACTGGGTTTTACAAACCTGTGA	TCCTT C GCACGCCGTGGGAC	TC T GTGGCAGGACTCGC
The primer/probe binding regions of the seven DNA oligonucleotides ordered to test the program (109 base pairs in length, with regions between primers consistent with the sequences for each accession number. The black sequence (top row) represents the reference template (R); grey sequences represent the varying DNA templates, with black highlighted bases depicting primer/probe-template mismatch sites. FMDV serotypes were as follows: JX040500 (O); KC440884 (Southern African Territories 2); AY593802 (A); KC440883 (O); AY593812 (O); KF112882 (O); HM191257 (O).			

**Table 4:** The effect of mismatches on ΔC_T_ calculated from the linear model analysis.

Factor	Mismatch Type	ΔC_T_	SE	t Value	*p* Value
Primer	% mismatch (forward/reverse combined) *	0.87	0.02	39.39	<0.001
	(minimum of 82.05% match is required [combined % for the pair])				
	nt 1 mismatch (type 1)	1.64	0.24	6.86	<0.001
	2× nt 1 mismatch (type 1)	4.88	0.81	6.02	<0.001
	nt 1 mismatch (type 2)	4.10	0.34	12.03	<0.001
	2× nt 1 mismatch (type 2)	8.71	1.25	6.97	<0.001
	nt 2 mismatch (type 1)	0.90	0.36	2.51	0.012
	2× nt 2 mismatch (type 1)	3.32	0.76	4.40	<0.001
	nt 2 mismatch (type 2)	3.44	0.39	8.82	<0.001
	2× nt 2 mismatch (type 2)	6.13	1.06	5.79	<0.001
	nt 3-4 mismatch (type 1)	1.07	0.40	2.69	0.007
	2× nt 3-4 mismatch (type 1)	2.14 **			
	nt 3-4 mismatch (type 2)	2.99	0.34	8.79	<0.001
	2× nt 3-4 mismatch (type 2)	4.83	2.97	1.63	0.105
	Maximum of two mismatches can be tolerated in the 3′-ends (within or between primers)				
Probe	% mismatch	0.50	0.03	18.35	<0.001
	(minimum of 85.00% match is required)				
(nt) nucleotide; (ΔC_T_) change in cycle threshold; (SE) standard error. For multiple mismatches, the linear model was able to calculate the effect of having the same type of mutation in both the primers (2×), if two mismatches were present but different the linear model calculated the additive/dampening effect: two 3′-end primer mismatches (ΔC_T_: −0.27 [2dp]); one primer and one probe mismatch (ΔC_T_: +0.43 [2dp]). Mismatches were grouped as one of two types: (type 1) purine-pyrimidine mismatch (G-T; C-A: minor conformational change in the primer/probe-template duplex); (type 2) purine-purine or pyrimidine-pyrimidine mismatch (G-A; A-A; G-G; C-T; T-T; C-C: major conformational change in the primer/probe-template duplex). One linear model looked primer-template mismatches; a second linear model was used to look at probe-template mismatches. * If (for example) a type nt 1 mismatch was present, the percentage mismatch ΔC_T_ would be calculated and an additional nt 1 mismatch ΔC_T_ penalty added. ΔC_T_, SE, and t value given to 2 decimal places. ** Insufficient oligos to calculate with accuracy, therefore GoPrime calculates this based on ΔC_T_ of nt 3–4 mismatch (type 1) × 2.					

## Supplementary Materials

The following are available online at https://www.mdpi.com/2076-0817/9/4/303/s1, Table S1: Statistical analyses to determine linear model variables; Figure S2: GoPrime example output; Table S3: Predicted and observed ΔCT and ΔLOD for the linear DNA templates representing FMDV field isolates for testing GoPrime predictions; Figure S4: Using GoPrime to predict the likely targets of four foot-and-mouth disease virus (FMDV)-typing RT-PCR assays.
