# Supplementary material for: GoPrime: Development of an In Silico Framework to Predict the Performance of Real-Time PCR Primers and Probes Using Foot-and-Mouth Disease Virus as a Model
Source: Pathogens. 2020 Apr 20;9(4):303. doi: 10.3390/pathogens9040303 (PMC7238122; doi:10.3390/pathogens9040303)
Supplement: Supplementary file 1 [file pathogens-09-00303-s001.zip › pathogens-739809-supplementary/Supplementary data_1.docx]

**Supplementary data, Table S1** Statistical analyses to determine linear model variables

| Region | Templates [mismatch] | Tukey test *p*-value | Result |
| --- | --- | --- | --- |
| 3ʹ-end of primers | A: 45-47 (nt 1 and 2) | A-B: *p* < 0.001  A-C: *p* = 0.083  B-C: *p* = 0.307 | No significant difference present between bases 3 and 4 (grouped together in the linear model). Previously published in Stadhouders *et al.* (2010). |
|  | B: 42-44 (nt 1 and 3) |  |  |
|  | C: 51-53 (nt 1 and 4) |  |  |
| One-way analysis of variance (ANOVA) tests (with post-hoc Tukey tests) to determine which primer/probe-template mismatch positions gave statistically different changes in cycle threshold. (nt): nucleotide. Template numbers refer to the oligonucleotide sequence in Table 1. | | | |
