# Supplementary material for: GoPrime: Development of an In Silico Framework to Predict the Performance of Real-Time PCR Primers and Probes Using Foot-and-Mouth Disease Virus as a Model
Source: Pathogens. 2020 Apr 20;9(4):303. doi: 10.3390/pathogens9040303 (PMC7238122; doi:10.3390/pathogens9040303)
Supplement: Supplementary file 1 [file pathogens-09-00303-s001.zip › pathogens-739809-supplementary/Supplementary data_2.docx]

**Supplementary data, Figure S2.** GoPrime example output.

GoPrime provides the outputs as two separate text files (A) a simple analysis, which provides each sequence name against the predicted ΔC_T_ and ΔLOD, number of mismatches present and the likely amplicon length; (B) a more detailed analysis, including the position and orientation of each likely primer/probe target and number and the type of any mismatches present. This output is for the linear DNA templates representing naturally occurring FMDV field isolates (Figure 4)**.**

1. GoPrime Output: simple analysis. Fields are (i) Target sequence name; (ii) ΔC_T_; (iii) ΔLOD; (iv) forward primer ΔC_T_ and start/end co-ordinates of primer binding site on the target sequence, (v) probe ΔC_T_ and start/end co-ordinates of probe binding site on the target sequence; (vi) reverse primer ΔC_T_ and start/end co-ordinates of primer binding site on the target sequence; (vii) length of PCR product.

>JX040500 3.3 0.96 Fwd[CT=3.3]=2/23 Probe=[CT=0]=53/72 Rev=[CT=0]=108/92 106

>KC440884 4.46 1.3 Fwd[CT=4.46]=2/23 Probe=[CT=0]=53/72 Rev=[CT=0]=108/92 106

>AY593802 8.03 2.34 Fwd[CT=0]=2/23 Probe=[CT=2.5]=53/72 Rev=[CT=5.53]=108/92 106

>KC440883 5.53 1.61 Fwd[CT=0]=2/23 Probe=[CT=0]=53/72 Rev=[CT=5.53]=108/92 106

>AY593812 5.22 1.52 Fwd[CT=0]=2/23 Probe=[CT=0]=53/72 Rev=[CT=5.22]=108/92 106

>KF112882 2.5 0.73 Fwd[CT=0]=2/23 Probe=[CT=2.5]=53/72 Rev=[CT=0]=108/92 106

>HM191257 5.8 1.69 Fwd[CT=0]=2/23 Probe=[CT=2.5]=53/72 Rev=[CT=3.3]=108/92 106

1. GoPrime Output: detailed analysis. For the output provided (sequence JX040500), GoPrime identifies a single potential binding site for the forward primer (position 23), probe (position 72) and reverse primer (position 92). In each case, the percentage match between the primer/probe and template is reported (and for primers a percentage match across the pair), the total number of mismatches across the whole primer (“TotMis”) and the total mismatches in the 3’ end (last 4 nucleotides) of the primer (“Tot1-4”). A single potential forward/probe/reverse set is found (“1-[1]-1”) in the expected orientation (forward going forward in direction, reverse going in reverse direction), with the predicted ΔC_T_ of 3.3. This is a result of mismatches between the forward primer and the template, as the probe and reverse primer are perfect matches.

Evaluating seq 1 >JX040500

23 position is a candidate for 5'-Fwd-3'-fwd 95.45% 97.44% [%Match %MatchPair], 1 1 [TotMis Tot1-4]

72 position is a candidate for 5'-Probe-3'-probe 100% 100% [%Match %MatchPair], 0 0 [TotMis Tot1-4]

92 position is a candidate for 3'-Rev-5'-rev 100% 100% [%Match %MatchPair], 0 0 [TotMis Tot1-4]

1-[1]-1 Fwd-[Probe]-Rev individual candidate primer/probe positions found in expected orientation

0-[1]-0 Fwd-[Probe]-Rev individual candidate primer/probe positions found in opposite orientation

Set-1 RT-PCR Success Fwd=2/23 Probe=53/72 Rev=108/92

deltaCT=3.3 [fCT=3.3 pCT=0 rCT=0]
