# Supplementary material for: GoPrime: Development of an In Silico Framework to Predict the Performance of Real-Time PCR Primers and Probes Using Foot-and-Mouth Disease Virus as a Model
Source: Pathogens. 2020 Apr 20;9(4):303. doi: 10.3390/pathogens9040303 (PMC7238122; doi:10.3390/pathogens9040303)
Supplement: Supplementary file 1 [file pathogens-09-00303-s001.zip › pathogens-739809-supplementary/Supplementary data_3.docx]

**Supplementary data, Table S3.** Predicted and observed ΔC_T_ and ΔLOD for the linear DNA templates representing FMDV field isolates for testing GoPrime predictions.

|  | **Predicted ΔC_T_** | **Average ΔC_T_**  **(Excite^TM^ UF)** | **Average ΔC_T_**  **(SSIII)** | **Predicted ΔLOD** | **Average ΔLOD**  **(Excite^TM^ UF)** | **Average**  **ΔLOD (SSIII)** |
| --- | --- | --- | --- | --- | --- | --- |
| JX040500 | 3.30 | 1.39 | 1.18 | 0.96 | 0 | 0 |
| KC440884 | 4.46 | 5.79 | 6.28 | 1.30 | 1 | 1 |
| AY593802 | 8.03 | 8.43 | 7.77 | 2.34 | 2 | 2 |
| KC440883 | 5.53 | 6.65 | 6.60 | 1.61 | 2 | 2 |
| AY593812 | 5.22 | 4.84 | 2.61 | 1.52 | 1 | 0 |
| KF112882 | 2.50 | 3.39 | 2.12 | 0.73 | 0 | 0 |
| HM191257 | 5.80 | 4.87 | 3.06 | 1.69 | 1 | 1 |
| Evaluating GoPrime as a predictor of real-time PCR (rPCR) performance using naturally occurring sequence variations. Two kits were tested: Excite^TM^ UF 2x Master Mix and SuperScript™ III Platinum™ One-Step qRT-PCR Kit. For the observed results, results represent the average change in cycle threshold or limit of detection across all dilutions (10^6^-10^0^) of starting template. FMDV serotypes were as follows: JX040500 (O); KC440884 (Southern African Territories 2); AY593802 (A); KC440883 (O); AY593812 (O); KF112882 (O); HM191257 (O). All results are given to 2dp. | | | | | | |
