# Supplementary material for: GoPrime: Development of an In Silico Framework to Predict the Performance of Real-Time PCR Primers and Probes Using Foot-and-Mouth Disease Virus as a Model
Source: Pathogens. 2020 Apr 20;9(4):303. doi: 10.3390/pathogens9040303 (PMC7238122; doi:10.3390/pathogens9040303)
Supplement: Supplementary file 1 [file pathogens-09-00303-s001.zip › pathogens-739809-supplementary/Supplementary data_4.docx]

**Supplementary data, Figure S4.** Using GoPrime to predict the likely targets of four foot-and-mouth disease virus (FMDV)-typing RT-PCR assays.


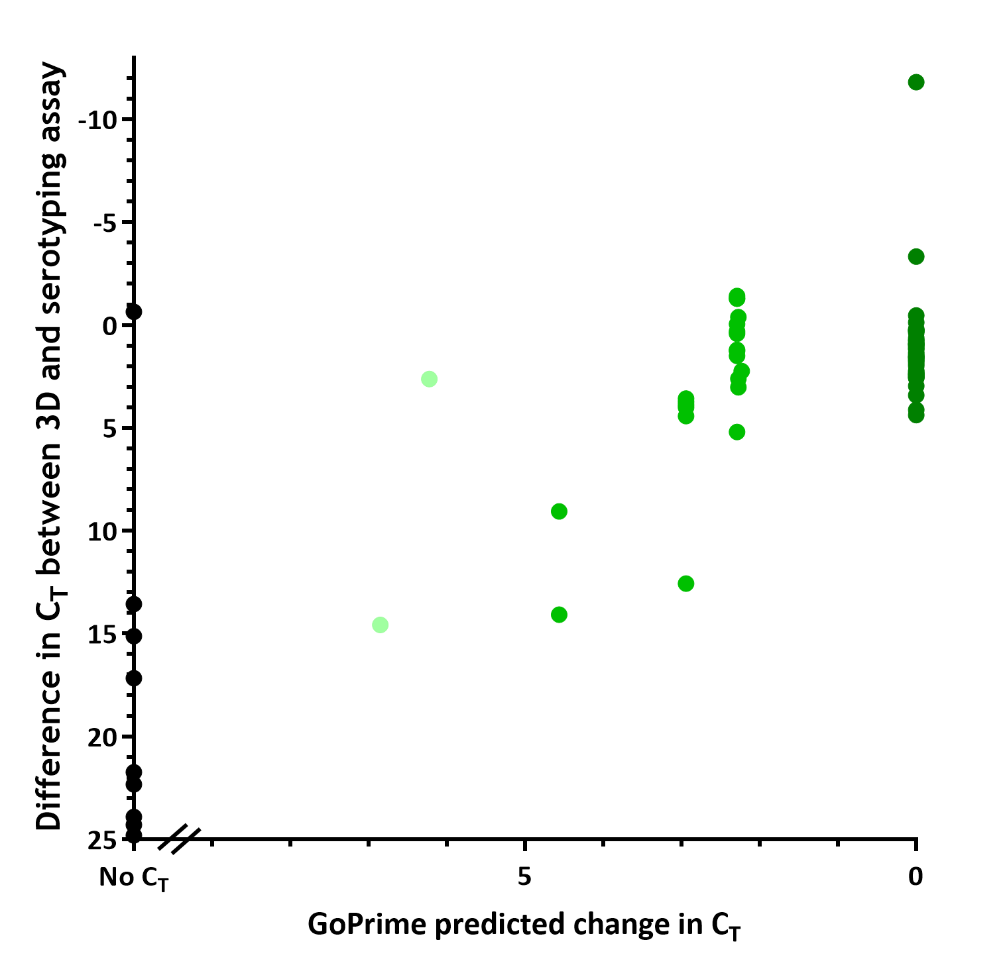


Four primer/probe sets from Bachanek-Bankowska *et al*. (2016) were analysed in GoPrime (serotype A; O; Southern African Territories [SAT] 1; SAT 2) and compared against the observed results. For this analysis, the observed ΔC_T_ was estimated by calculating the difference between the C_T_ value for the 3D assay (assumed perfect match) and serotype-specific assays. The colour scheme indicates the predicted results from GoPrime: (dark green) perfect primer/probe-template match; (mid-green) cycle threshold affected by up to a C_T_ of 5; (light green) cycle threshold affected by up to a C_T_ of 10; (black) sequence predicted not to amplify.

GoPrime is designed for use with real-time PCR, however was able to identify the likely positive targets for the four FMDV-typing RT-PCR assays. However, cross-reactivity between serotypes was not predicted and was evident in the published results. In order to improve accuracy, future experimental set ups should include analysis of the reverse transcription step (effect of mismatches between the RNA templates and primers) and further analysis of clinical samples across a broader genomic context.
